# Sensitivity, specificity, and predictive power of the “Brief Risk-resilience Index for SCreening,” a brief pan-diagnostic web screen for emotional health

**DOI:** 10.1002/brb3.76

**Published:** 2012-07-26

**Authors:** Leanne M Williams, Nicholas J Cooper, Stephen R Wisniewski, Justine M Gatt, Stephen H Koslow, Jayashri Kulkarni, Savannah DeVarney, Evian Gordon, Augustus John Rush

**Affiliations:** 1 BRAINnet Foundation71 Stephenson Street, Suite 400, San Francisco, California, 94105; 2 University of Sydney Medical School and Westmead Millennium InstituteSydney, New South Wales, 2145, Australia; 3 Brain ResourceLevel 12, 235 Jones Street, Ultimo, Sydney, 2007, Australia; 4 Brain Resource1000 Sansome Street, San Francisco, California, 94111; 5 Department of Epidemiology, University of Pittsburgh127 Parran Hall, Pittsburgh, Pennsylvania, 15261; 6 Duke-NUS Graduate Medical School Singapore8 College Road, Singapore, 169857

**Keywords:** Depression and anxiety, emotional well-being, Internet, mental health screen, risk and resilience, sensitivity and specificity

## Abstract

Few standardized tools are available for time-efficient screening of emotional health status across diagnostic categories, especially in primary care. We evaluated the 45-question Brief Risk-resilience Index for SCreening (BRISC) and the 15-question mini-BRISC in identifying poor emotional health and coping capacity across a range of diagnostic groups – compared with a detailed clinical assessment – in a large sample of adult outpatients. Participants 18–60 years of age (*n* = 1079) recruited from 12 medical research and clinical sites completed the computerized assessments. Three index scores were derived from the full BRISC and the mini-BRISC: one for risk (negativity–positivity bias) and two for coping (resilience and social capacity). Summed answers were converted to standardized *z*-scores. BRISC scores were compared with detailed health assessment and diagnostic interview (for current psychiatric, psychological, and neurological conditions) by clinicians at each site according to diagnostic criteria. Clinicians were blinded to BRISC scores. Clinical assessment stratified participants as having “clinical” (*n* = 435) or “healthy” (*n* = 644) diagnostic status. Receiver operating characteristic analyses showed that a *z*-score threshold of −1.57 on the full BRISC index of emotional health provided an optimal classification of “clinical” versus “healthy” status (sensitivity: 81.2%, specificity: 92.7%, positive predictive power: 80.2%, and negative predictive power: 93.1%). Comparable findings were revealed for the mini-BRISC. Negativity–positivity bias index scores contributed the most to prediction. The negativity–positivity index of emotional health was most sensitive to classifying major depressive disorder (100%), posttraumatic stress disorder (95.8%), and panic disorder (88.7%). The BRISC and mini-BRISC both offer a brief, clinically useful screen to identify individuals at risk of disorders characterized by poor emotion regulation, from those with good emotional health and coping.

## Introduction

Emotional dysregulation is a feature of multiple psychiatric, psychological, and neurological conditions, and conversely, effective emotional regulation characterizes positive well-being, coping, and resilience. Our aim was to use these features to identify a broad screen for poor versus good emotional health across diagnostic and community samples.

Approximately 60% of patients who have psychiatric and neurological disorders seek care from primary care physicians (Regier et al. 1978; Ezzati-Rice and Rohde 2008). Clinicians who are not psychiatric or neurological specialists are increasingly expected to serve roles in early identification, management, and ultimately prevention of these disorders. (Druss et al. 2010). To support these roles, there is demand for a quick screen that can be applied across broad populations and provide immediate feedback. Ideally, such screening tools would be time effective for both physician – given typical heavy patient loads – and patient – picking up a broad set of conditions earlier and more effectively. They would provide an objective and accurate way to identify individuals at risk of psychiatric and neurological conditions, and factor in behaviors which contribute to resilience and capacity to cope. Furthermore, they would provide immediate feedback on case identification via automated reporting.

There is currently a dearth of standardized tools that provide a broad screen of this kind. At the population level, mental health-related disorders go unidentified and thus untreated in 50–65% of cases (Nielson and Williams 1980; Kessler et al. 1985; Schulberg et al. 1985; Katon 1987; Barret et al. 1988; Borus et al. 1988; Schulberg and Burns 1988; Andersen and Harthorn 1989; Ormel et al. 1991; Rydon et al. 1992). Of the available self-report screening scales that could be considered brief and comprising sound psychometric properties, the focus is on screening for a particular diagnosis (Mulrow et al. 1995). For example, the Patient Health Questionnaire-9 item (PHQ-9) screens specifically for diagnostic criteria of depressive disorder (Kroenke et al. 2010), and the Quick Inventory of Depressive Symptoms – Self-Report (QIDS-SR) assesses the severity of symptoms in major depressive disorder (Rush et al. 2003). Other scales are focused on health-related outcomes. For example, the Medical Outcomes Study Short Form (SF-36; Ware and Sherbourne 1992) and its even shorter version (SF-12) are a psychometrically sound survey designed to assess quality-of-life outcomes across diagnoses. It is not intended as a screening tool. Other pan-diagnostic scales with robust psychometric qualities are focused on outcomes for a related set of diagnoses. For example, the OASIS is a brief self-report scale for assessing frequency of anxiety, intensity of anxiety, behavioral avoidance, and functional impairment associated with anxiety to determine symptom and functional outcomes across diagnoses of anxiety disorder (Campbell-Sills et al. 2009).

The BRISC is designed to address gaps in these available tools. First, it provides a quick screen for emotional health relative to a wide spectrum of diagnoses and healthy people, which is not available in currently available instruments. This enables identification of cases at *risk* of poor mental and neurological health across various disorders and practice settings. Second, it includes measures of *coping* to inform the triage of those most at risk and coping poorly versus those who are resilient and coping well. This information is also not provided by available instruments. The BRISC has been validated against other self-report measures of emotional health, functional outcome measures, and biological susceptibility factors (for details, see Methods). It is designed to provide a time- and cost-effective screen, delivered via the web, with immediate reporting on results.

This study was designed to evaluate the sensitivity, specificity, and predictive power of the 45-item BRISC and the 15-item “mini-BRISC” in distinguishing clinical versus healthy status across a range of disorders in a large sample of adult outpatients and healthy volunteers. BRISC scores were compared with a detailed assessment of clinical status.

## Method

### The BRISC

The BRISC was developed and validated within a framework called the “INTEGRATE model”, which draws on psychiatric, psychological, physiological, and neuroscience theories (Gordon et al. 2008; Williams et al. 2008). It is designed to measure, by self-report, the spectrum of good versus poor self-regulation of emotional functions, which underlies mental health and has a basis in neurobiology.

The BRISC measures three core domains: negativity bias, emotional resilience, and social skills. Negativity bias represents hypersensitivity to stress and the expectation of negative outcomes, which elevate the risk for poor brain health (Wichers et al. 2007; Williams et al. 2009, 2010). Positivity Bias is the opposing tendency and quantifies a lack of negativity bias and an expectation of positive and/or neutral outcomes. Emotional resilience is the capacity for self-efficacy. It is premised in the notion that having a “thick skin” (or emotional resilience) may offset poor mental functioning and facilitate good functioning. Social skills is the capacity to engage socially and seek support. These attributes contribute to the ability to cope with poor mental functioning and to facilitate good functioning. Development of the BRISC followed a stepwise process which is detailed in its manual (Brain Resource Ltd publishers 2010). The five main validation steps are summarized below:

#### Construct validation of content domains

These three domains were validated by principal components analyses of an initial pool of 93 items (Rowe et al. 2007; Williams et al. 2008). Factor analysis confirmed the presence of domains reflecting negativity–positivity bias, emotional resilience, and social skill capacity in a healthy volunteer sample of 1000 individuals who spanned nine decades in age (Rowe et al. 2007; Williams et al. 2008). Using regression analysis, we reduced the number of items loading on these factors to the core 45 items needed to predict them (Wichers et al. 2007). This structure was replicated in an independent sample of 1557 (Brain Resource Ltd publishers 2010).

#### Face validation

To achieve face validity, the phrasing of the 45 items was slightly adjusted so that the tense and format of the questions in each item were consistent, without affecting the content of the question. The questions are listed in Appendix 1

#### Construct validation using self-report measures of regulation

*Sample:* A community sample of 55 healthy volunteers participated in this component of the study (mean age 29.13 ± 8.80 years, range: 19–55 years; 76.4% female). Exclusion criteria were Axis 1 criteria for psychiatric disorder (assessed using the Somatic and Psychological Health Report Questionnaire, SPHERES-12; Hickie et al. 2001), Patient Health Questionnaire for indicators of eating disorder (Kroenke et al. 2010), indicators of neurological disorder (assessed using items from the mental status examination; Trzepacz and Baker 1993), and indicators of alcohol and/or substance dependence assessed using the AUDIT and Fagerstrom nicotine dependency questionnaire (Heatherton et al. 1991; Bush et al. 1998).

Assessments:

- Emotion Regulation Scale (Gross and John 2003): To assess the capacity to regulate one's emotions in terms of both reappraisal and suppression strategies
- Internal Control Index (ICI; Duttweiler 1984): To assess internal locus of control related to the belief that reinforcement is contingent on one's own behavior, related to self-confidence and autonomy

*Validation outcomes:* We conducted correlation analyses between the BRISC scales and the ERQ and ICI, using a corrected *P*-value of 0.005. Results demonstrated convergent construct validation for each BRISC scale as follows:

- Negative correlations between lower negativity–positivity bias and higher scores on ICI components of internal control (*r* = −0.51, *P* < 0.0001) and self-confidence (*r* = −0.51, *P* < 0.0001)
- Positive correlations between higher emotional resilience and higher scores on the ICI components of internal control (*r* = 0.39, *P* = 0.003) and self-confidence (*r* = 0.39, *P* = 0.003)
- Positive correlations between higher social skills and higher scores on the ICI perceived control component (*r* = 0.40, *P* = 0.003) and the ERQ reappraisal strategy component (*r* = 0.56, *P* < 0.0001)

#### Construct validation of the negativity bias measures using genetic, autonomic, and brain imaging measures

*Sample:* Three hundred and three healthy volunteers of European ancestry (mean age 32.92 ± 10.73 years, range: 18–54 years, 49.5% female) took part and completed the BRISC, heart rate recording, and genotyping (Williams et al. 2009, 2010). Of these, matched subsets of 39 and 46 also completed functional magnetic resonance imaging (fMRI) (Williams et al. 2009, 2010).

*Assessments:* Heart rate and fMRI were recorded during a facial emotion viewing task, under both conscious and nonconscious conditions. DNA was extracted from cheek swab samples and genotyped for the polymorphism of the serotonin transporter gene (5-HTT-LPR) and COMT Val108/158Met genotypes (for details of Methods, Williams et al. 2009, 2010).

*Validation outcomes:* Greater fear reactivity indexed by both heart rate and activation of brainstem, amygdala, and medial prefrontal cortex circuitry was associated with greater negativity relative to positivity bias. This association was pronounced in individuals with the “risk” alleles, 5 HTT-LPR Short and COMT Met. The findings indicate that a higher negativity bias is underpinned by genetic and fear circuitry susceptibility for emotional disorder.

#### Ecological validation with real-world functional capacities

The association between the full BRISC and proxy measures of real-world functional outcomes was established in the same sample of 55 participants used to assess construct validation against the ERQ and IC. These proxy measures included the following:

- Quality of Life assessed by the World Health Organizations Qualify of Life scale, brief version (WHOQOL-BREF) scale (World Health Organization Group 1998)
- Satisfaction with Life Scale (SWLS; Diener et al. 1985)
- Work productivity, in terms of both absenteeism (hours absent from work) and presenteeism (performance level), assessed using the Health and Work Performance Questionnaire (HPQ; Kessler et al. 2003)

*Validation outcomes:* Correlation analyses between the BRISC scales and the WHOQOL-BREF, SWLS, and HPQ, at a corrected *P*-value of 0.01, demonstrated the following associations:

- Negative correlations between lower negativity–positivity bias and higher WHOQOL-BREF psychological (*r* = −0.50, *P* < 0.0001) and social relationships (*P* = −0.39, *P* = 0.003) components of quality of life, and the presenteeism component of the productivity on the HPQ (*r* = −0.39, *P* = 0.01)
- Positive correlations between higher emotional resilience and higher scores on the WHOQOL-BREF psychological component (*r* = 0.52, *P* < 0.0001) and satisfaction with life on the SWLS (*r* = 0.34, *P* = 0.01)
- Positive correlations between higher social skills and higher scores on the WHOQOL-BREF components of physical health (*r* = 0.45, *P* = 0.001) and environment (*r* = 0.56, *P* < 0.0001), satisfaction with life (*r* = 0.42, *P* = 0.001) and presenteeism on the HPQ (*r* = 0.37, *P* = 0.008)

### Study research sites

Participants were recruited from 12 medical research or clinical research sites. These sites agreed to collaborate as partners with Brain Resource to evaluate brain health in patients using a standardized set of assessments and contribute the data to a centralized library (the Brain Resource International Database). The medical research sites were located in universities with teaching hospital outpatient clinics in psychiatry and psychology. The clinical research sites were multidisciplinary outpatient clinics that offer brain health assessment and treatment services (such as EEG testing) for any medical condition. Expert clinicians at each site completed diagnostic interviews and were blinded to the results of the BRISC and other self-report assessments.

### Recruitment

This retrospective study recruited participants through advertising and self-referral. Inclusion criteria were in regard to the capacity to undergo a computerized test: reading at Year 5 level (equivalent to Year 6 in England and fifth grade in the United States), normal (or corrected to normal) vision, and ability to use a keyboard. The protocol received independent ethics committee or institutional review board approval before recruitment of participants. All participants signed and dated an approved informed consent form. Where participants consented, these data have also been made available for open sharing and secondary analysis by the research community (Gordon et al. 2005, 2008). All research is in compliance with the Code of Ethics of the World Medical Association (Declaration of Helsinki).

### Main measures

#### The assessment of behavioral health status

**Figure 1:**
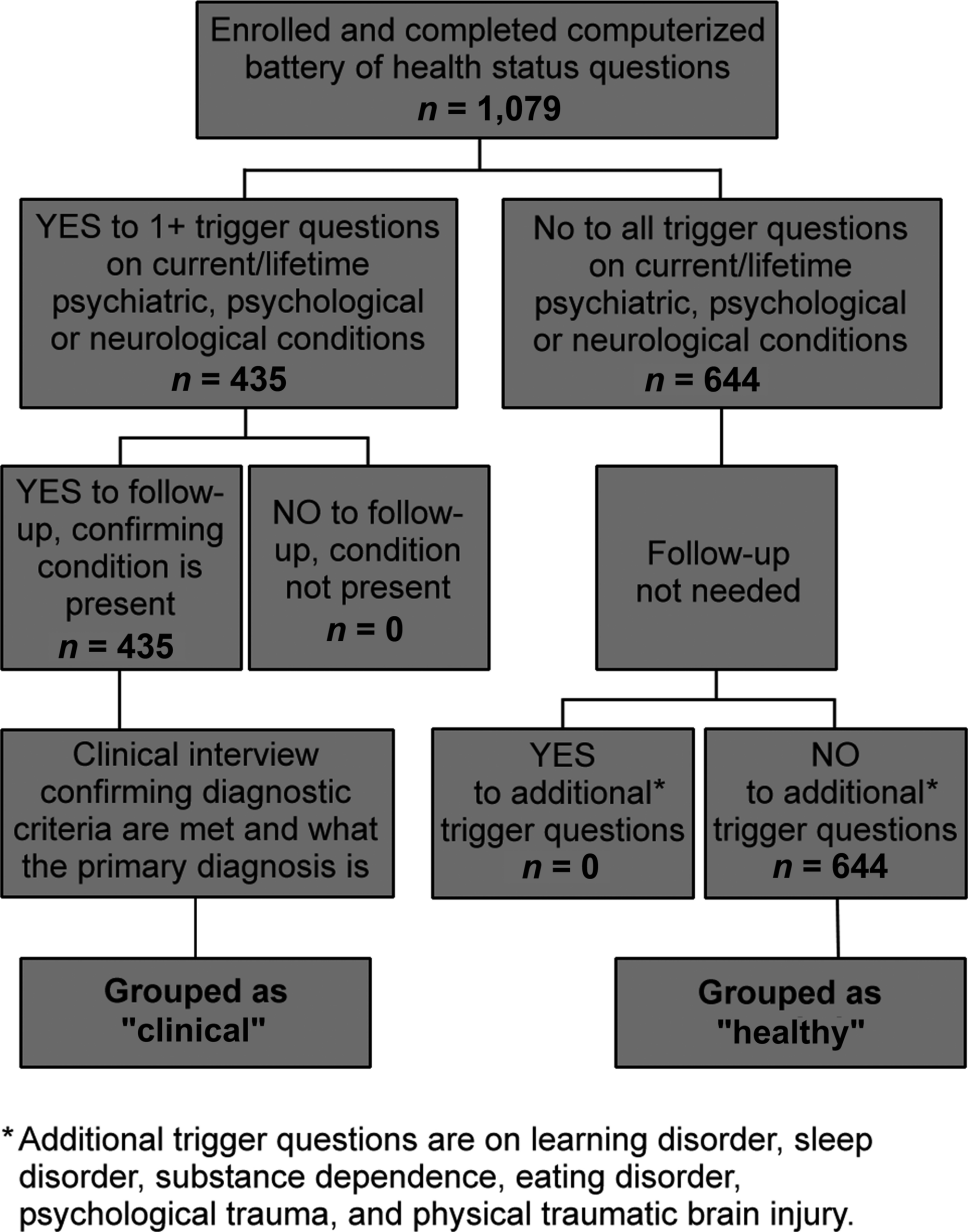
Summary of the criteria for independent classification of “good” versus “poor” brain health status.

At the testing site, participants first completed a computer battery of detailed questions to provide an independent determination of behavioral health status. This assessment comprised established items to assess current or lifetime psychiatric and neurological conditions (Table 1). Stepwise stratification logic was used to determine “clinical” versus “healthy” behavioral health according to the criteria summarized in Figure 1.

**Table 1:** Summary items used in the independent assessment of clinical versus healthy status

Number and type of items*	Areas assessed	Source of items
2 Trigger items (yes, no) 8 Follow-up	Current or lifetime diagnosis of psychiatric and psychological disorder If yes, the nature of the disorder, whether it is current, what the previous history is, duration, and treatment	Mental status examination (Trzepacz and Baker 1993)
12 items	Current Axis 1 criteria for common mental disorders, focused on depressive and anxiety disorders	Somatic and Psychological Health Report Questionnaire (SPHERE-12) screening tool (Hickie et al. 2001)
2 Trigger items (yes, no) 8 Follow-up	Current or lifetime diagnosis of neurological disorder If yes, the nature of the disorder, whether it is current, what the previous history is, duration, and treatment	Mental status examination
1 Trigger item (yes, no) 5 Follow-up	Experience of learning disorder/dyslexia If yes, extent of disruption at school, and whether or not dyslexia diagnosis was given	Mental status examination
3 Trigger items (yes, no) 16 Follow-up	Sleep impairment (past month) If yes, extent of impairment and criteria for sleep apnea	Mental status examination Maislin Sleep Apnea Index (Maislin et al. 1995)
1 Trigger item (yes, no) 7 Follow-up	Regular disordered eating If yes, items to cover criteria for anorexia and bulimia nervosa	Mental status examination Patient Health Questionnaire (Spitzer et al. 1999)
3 Trigger items (yes, no) 21 Follow-up	Regular use of alcohol or other recreational drugs of dependence and current medication If yes, AUDIT to assess alcohol dependence, Fagerstrom Test for Nicotine Dependence and WHO criteria for drug dependence for marijuana and other major categories of illicit drugs	Mental status examination AUDIT (Bush et al. 1998) Fagerstrom Test for Nicotine Dependence (Heatherton et al. 1991)
1 Trigger item (yes, no) 30 Follow-up	Psychological trauma If yes, items for DSM–IV criterion A stressors for PTSD	Mental status examination DSM–IV items for criterion A stressors (Breslau and Kessler 2001)
1 Trigger item (yes, no) 3 Follow-up	Major surgery to brain or spine If yes, nature of surgery	Mental status examination
1 Trigger item (yes, no) 7 Follow-up	Physical trauma If yes, nature of physical injury causing substantive loss of consciousness	Mental status examination
1 Trigger item (yes, no) 12 Follow-up	Current medication If yes, type, reason, dose, frequency for up to three medications	Mental status examination

* These items are implemented in web questionnaire called “WebQ”.

#### The BRISC

After the assessment of behavioral health status, yet in the same testing session, participants completed the 45-question BRISC (Appendix 1) via computer, which took about 10 min to complete. The results provided one score for risk (negativity bias) and two scores for coping (emotional resilience and social skills; Rowe et al. 2007; Williams et al. 2008). As indicated in Appendix 1, the 15-question mini version of the BRISC is made up of the five highest-loading BRISC items for each of the core content domains: negativity bias, emotional resilience, and social skills.

Responses to each BRISC question were made on a scale of 1–5, with 5 representing higher functioning (less risk, better coping). We summed the responses for negativity bias, for emotional resilience, and for social skills (raw scores are shown in Appendix 2 for the 45-question BRISC and Appendix 3 for the mini-BRISC). These summed responses were converted to standardized *z*-scores, using norms in 1317 nonclinical participants established for the BRISC (Rowe et al. 2007). The results of both the assessment of health status and the BRISC were not provided to the participant or the investigator at the time of testing.

### Diagnostic interview

The clinicians at each site also completed a semistructured diagnostic interview for each participant which included the current status of any psychiatric, psychological, or neurological disorder. The interview provided confirmation of the disorder against diagnostic criteria, as well as the nature of the primary diagnosis. Clinics were psychiatrists, neurologists, and clinical psychologists.

### Methods of analysis

Analyses were undertaken using *z*-scores for negativity bias, emotional resilience, and social skills for the full BRISC and the mini-BRISC. Pearson correlations were used to examine associations between the three BRISC core content domain scores.

Receiver operating characteristic (ROC) curves were then generated using the “Epi” package from the statistical analysis program “R” version 2.10.1 (http://www.r-project.org/; Ihaka and Gentleman 1996). The goal of the ROC curves was to identify the optimal *z*-score cutpoint at which BRISC scores classified participants who were independently identified as positive for one or more psychiatric-neurological disorders (clinical) versus those identified as negative for these disorders (healthy). The optimal cutpoint was determined algorithmically to maximize sensitivity plus specificity. This threshold was annotated on these curves with a summary of classification performance. A priori *z*-score thresholds of −0.5, −1.0, −1.5, and −2.0 were also marked on each ROC curve to provide a context for the interpretation of the optimal threshold. The area under the curve (AUC) statistic was also generated in each case, where 1.0 is the maximum possible value. Sensitivity, specificity, positive predictive power, and negative predictive power were tabulated for the results at the optimal and a priori *z*-score thresholds.

## Results

### Characteristics of sample

From March 2005 through December 2009, 1079 participants (mean age = 37.0 years; range: 18–60 years, 51.8% female) completed the assessment of behavioral health status, the full 45-question BRISC, and the clinician-administered diagnostic interview. This sample represented a dataset without missing or indeterminate data. Overall, 644 participants were identified as being of “healthy” status as they answered “no” to all trigger questions. The remaining 435 participants were identified as being of “clinical” status as they answered “yes” to one or more of the trigger questions. The clinical diagnostic interview confirmed that all 435 met diagnostic criteria for a primary psychiatric, psychological, or neurological disorder. Of these 435, 260 met criteria for a primary depressive or anxiety disorder, including major depressive disorder (128, 29.4%), posttraumatic stress disorder (79, 18.2%), and panic disorder (53, 12.2%). Other disorders were traumatic brain injury (86, 19.8%), mild cognitive impairment (48, 11.0%), and psychosis (41, 9.4%; specified as first onset by clinicians based on no prior episodes and being within 3 months of first contact with the health service).

### Full BRISC

In the total sample (*n* = 1079), negativity–positivity bias scores correlated negatively and significantly with both emotional resilience (*r* = −0.499; *P* < 0.0001) and social skills (*r* = −0.279; *P* < 0.0001; Table 2). These correlations are consistent with the theoretical basis of the BRISC: that the marker of risk (negativity bias) will be inversely related to markers of coping (emotional resilience and social skills). Emotional resilience and social skills were found to have a significant overlap (*r* = 0.312; *P* < 0.0001). The degree of overlap is consistent with these markers, reflecting partially separable types of protective factors.

**Table 2:** Correlations between scores on the 45-question BRISC and 15-question mini-BRISC*

Samples	Negativity bias	Emotional resilience	Social skills
45-Question BRISC			
Total sample (*n* = 1079)			
Negativity bias	1.000	−.499†	−.297†
Emotional resilience		1.000	.312†
Social skills			1.000
Clinical status (*n* = 435)			
Negativity bias	1.000	−.522†	−.316†
Emotional resilience		1.000	.375†
Social skills			1.000
Healthy status (*n* = 644)			
Negativity bias	1.000	−.400†	−.131†
Emotional resilience		1.000	.242†
Social skills			1.000
15-Question BRISC			
Total sample (*n* = 1079)			
Negativity bias	1.000	−.330†	−.104†
Emotional resilience		1.000	.209†
Social skills			1.000
Clinical status (*n* = 435)			
Negativity bias	1.000	−.341†	−.115†
Emotional resilience		1.000	.239†
Social skills			1.000
Healthy status (*n* = 644)			
Negativity bias	1.000	−.141†	.072
Emotional resilience		1.000	.169†
Social skills			1.000

* Correlations calculated the Pearson correlation coefficient.

† *P* < 0.0001.

#### ROC analyses

**Figure 2:**
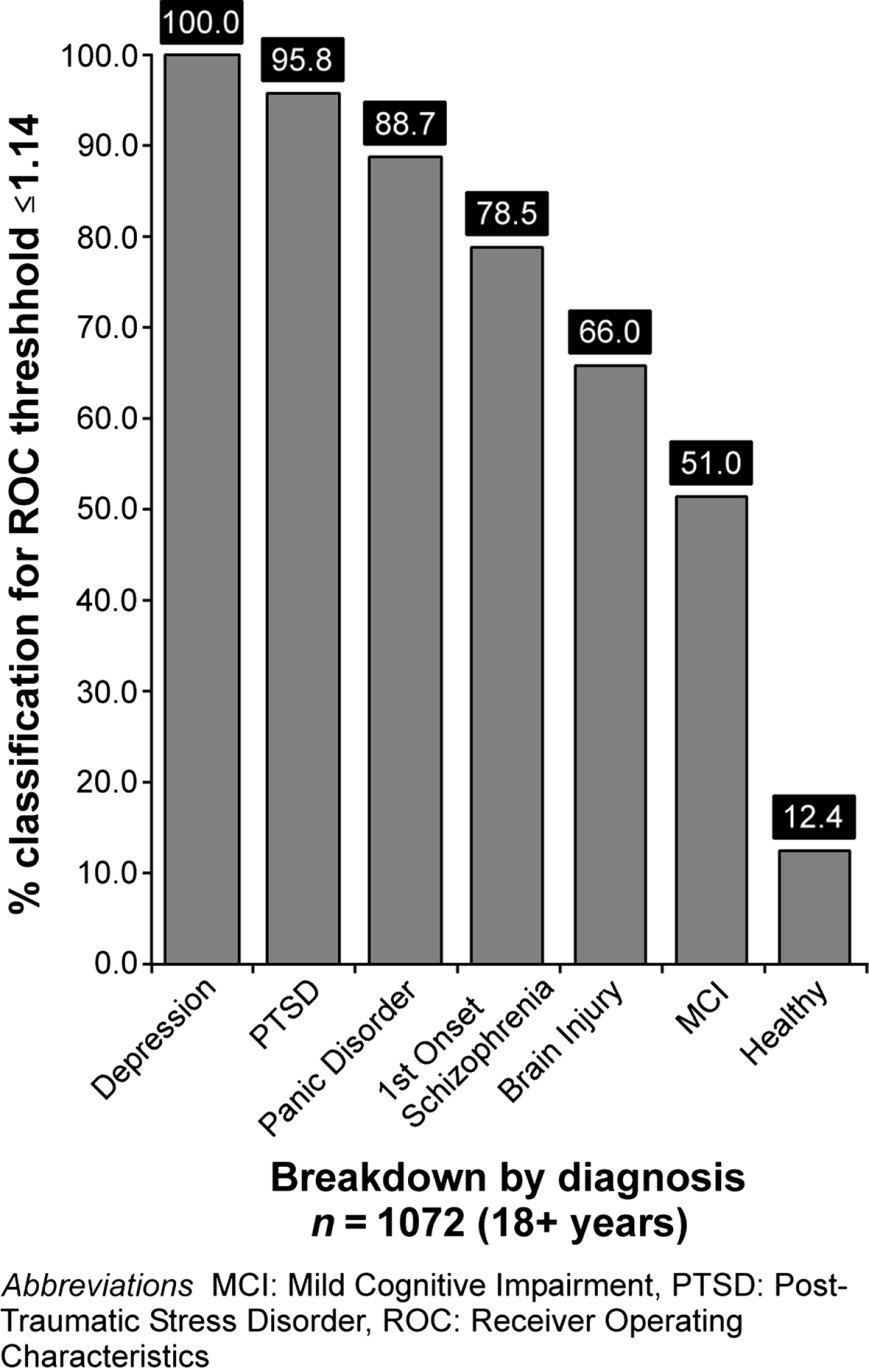
45-Item BRISC. Breakdown of classification by diagnosis for negativity bias using the ROC determined threshold.

In ROC analyses, negativity bias made the largest contribution to classification. Figure 2 shows the breakdown of clinically confirmed diagnoses for negativity bias in the “clinical” group. Sensitivity of the BRISC was highest for depression, posttraumatic stress disorder, and panic disorder, followed by psychosis, brain injury, and mild cognitive impairment.

Table 3 shows the ROC curve analysis results across negativity bias, emotional resilience, social skills, and combined total scores for the 45-item BRISC.

**Table 3:** Summary of sensitivity, specificity, and positive and negative predictive power of the 45-question BRISC scores at *z*-score thresholds of −2, −1.5, −1, and −0.5 and ROC determined optimal score

BRISC scores	*z*-Score thresholds				
Negativity bias					
	−2SD threshold	−1.5SD threshold	−1SD threshold	−0.5SD threshold	−1.14SD ROC threshold
Sensitivity (%)	91.4	86.4	78.9	67.4	84.9
Specificity (%)	71.9	84.9	91.9	97.4	87.6
Positive predictive power (%)	53.3	66.8	77.5	90.0	70.7
Negative predictive power (%)	96.0	94.7	92.5	89.5	94.3
Emotional resilience					
	−2SD threshold	−1.5SD threshold	−1SD threshold	−0.5SD threshold	−0.43SD ROC threshold
Sensitivity (%)	64.8	52.4	41.3	28.1	69.3
Specificity (%)	72.4	85.5	92.7	96.9	70.0
Positive predictive power (%)	54.5	64.9	74.2	82.4	54.1
Negative predictive power (%)	80.1	77.9	75.6	72.5	81.7
Social skills					
	−2SD threshold	−1.5SD threshold	−1SD threshold	−0.5SD threshold	−0.50SD ROC threshold
Sensitivity (%)	54.6	36.8	22.5	15.7	54.6
Specificity (%)	68.1	83.2	94.1	97.9	68.1
Positive predictive power (%)	37.7	43.6	57.3	72.1	37.7
Negative predictive power (%)	80.9	78.8	77.4	76.7	80.9
Combined score					
	−2SD threshold	−1.5SD threshold	−1SD threshold	−0.5SD threshold	−1.57SD ROC threshold
Sensitivity (%)	69.6	81.6	87.2	92.0	81.2
Specificity (%)	96.3	91.9	83.6	70.7	92.7
Positive predictive power (%)	87.4	78.7	66.0	53.4	80.2
Negative predictive power (%)	89.6	93.1	94.6	96.0	93.1

*z*-Score thresholds are expressed in standard deviations (SD). Results are reported for scores on negativity bias, emotional resilience, social skills, and combined total.

For the negativity bias score, the optimal *z*-score threshold for distinguishing clinical status was −1.14. This threshold was both sensitive (84.9%) and specific (87.6%) in classifying the clinical versus healthy groups. In addition to good positive predictive power at this threshold (70.7%), there was also high negative predictive power (94.3%; Table 3). The AUC value of 0.92 indicated a very high discrimination, reflective of overall accuracy.

Emotional resilience scores revealed a lower optimal threshold of *z* = −0.43 for distinguishing clinical from healthy status. Sensitivity was at 69.3% and specificity was at 70.0%. The results suggested that these scores contribute most to negative predictive power (81.7%) for supporting decisions about confirming good emotional health (Table 3). Overall accuracy was high (AUC was 0.75).

Social skills scores had an optimal threshold of *z* = −0.50 for classifying clinical from healthy groups. Sensitivity was at 54.6% and specificity was at 68.1%. Results for these scores suggest that they contribute most to negative predictive power (80.9%) relevant to the confirmation of healthy status (Table 3). These scores contributed to a good overall accuracy (AUC was 0.64).

When ROC analysis was run for the three BRISC scores combined, both positive and negative predictive power were maximized (Table 3). The optimal threshold was *z* = −1.57 for the combined scores, with a sensitivity of 81.2%, specificity of 92.7%, positive predictive power of 80.2%, and negative predictive power of 93.1%. These values generated a high overall accuracy (AUC of 0.93).

### Mini-BRISC

Correlations for the mini-BRISC showed very nearly the same pattern of associations for the total sample, and for the clinical and healthy groups, as were found with the full BRISC. The only exception was the lack of a significant inverse association between negativity bias and social skills for the “clinical” participants (Table 2).

#### ROC analyses

Table 4 summarizes the ROC curve analysis results for the 15-item BRISC. The mini-BRISC showed a very similar pattern of classification to the full BRISC. For the 5-item negativity bias score, the optimal threshold was *z* = −1.34, with a sensitivity of 79.9%, specificity of 89.2%, positive predictive power of 72.2%, and negative predictive power of 92.7% (Table 4). Overall accuracy remained very high (AUC of 0.92).

**Table 4:** Summary of sensitivity, specificity, and positive and negative predictive power of the 15-question mini-BRISC scores at *z*-score thresholds of −2, −1.5, −1, and −0.5 and ROC determined optimal score

BRISC scores	*z*-Score thresholds				
Negativity bias					
	−2SD threshold	−1.5SD threshold	−1SD threshold	−0.5SD threshold	−1.34SD ROC threshold
Sensitivity (%)	89.6	84.9	77.1	69.2	79.9
Specificity (%)	71.6	83.6	90.7	94.7	89.2
Positive predictive power (%)	52.6	64.6	74.4	82.1	72.2
Negative predictive power (%)	95.1	94.0	91.8	89.7	92.7
Emotional resilience					
	−2SD threshold	−1.5SD threshold	−1SD threshold	−0.5SD threshold	−0.95SD ROC threshold
Sensitivity (%)	9.3	21.4	36.8	53.9	47.4
Specificity (%)	98.7	96.2	88.6	72.2	83.3
Positive predictive power (%)	72.2	66.7	53.4	40.7	51.1
Negative predictive power (%)	75.5	77.6	79.9	81.6	81.2
Social skills					
	−2SD threshold	−1.5SD threshold	−1SD threshold	−0.5SD threshold	−0.61SD ROC threshold
Sensitivity (%)	44.6	27.5	17.5	12.1	43.6
Specificity (%)	68.8	85.5	94.3	97.1	71.1
Positive predictive power (%)	33.6	40.1	52.1	59.6	34.8
Negative predictive power (%)	77.9	76.9	76.4	75.8	78.7
Combined score					
	−2SD threshold	−1.5SD threshold	−1SD threshold	−0.5SD threshold	−1.31SD ROC threshold
Sensitivity (%)	69.2	77.2	83.6	89.2	80.0
Specificity (%)	94.7	91.0	84.1	73.2	89.3
Positive predictive power (%)	82.7	75.9	65.9	54.9	73.3
Negative predictive power (%)	89.3	91.6	93.3	94.8	92.4

*z*-Score thresholds are expressed in standard deviations (SD). Results are reported for scores on negativity bias, emotional resilience, social skills, and combined total.

The 5-item emotional resilience score showed an optimal threshold of *z* = −0.95. The results suggested that this score contributes most to specificity (83.3%) and negative predictive power (81.2%) for supporting decisions about confirming healthy status, rather than sensitivity to a clinical condition (Table 4). Accuracy was retained at a similarly high level to that for the full BRISC (AUC of 0.69).

For the 5-item social skills score, the optimal threshold was *z* = −0.61. The results suggest that this score also contributes most to specificity (71.1%) and negative predictive power (78.7%) for classifying good brain health (Table 4). Overall accuracy remained in the moderate to high range (AUC of 0.58).

For the three mini-BRISC scores combined, both positive and negative predictive power were maximized, as they were for the 45-question version (Table 4). The optimal threshold was *z* = −1.31 for the combined scores, with a sensitivity of 80.0%, specificity of 89.3%, positive predictive power of 73.3%, and negative predictive power of 92.4%. Overall accuracy was similarly high (AUC of 0.92).

## Discussion

**Figure 3:**
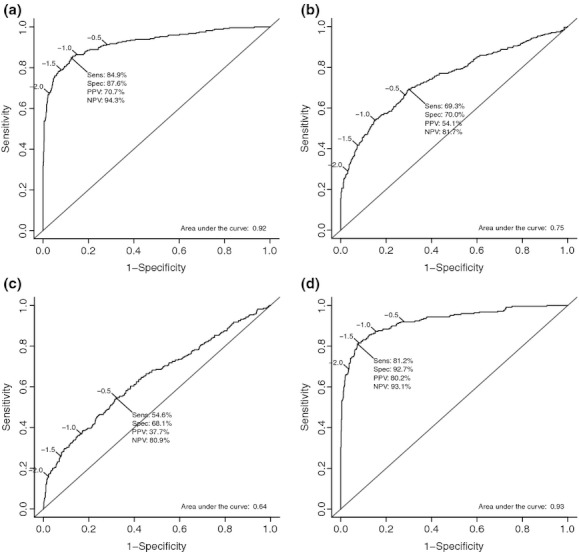
Receiver operating curve results for the 45-item BRISC, for negativity bias (a), emotional resilience (b), social skills (c), and all three scores combined (d).

**Figure 4:**
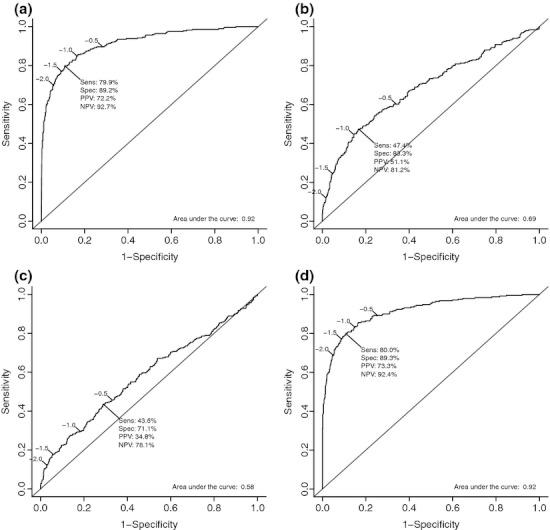
Receiver operating curve results for the 15-item BRISC, for negativity bias (a), emotional resilience (b), social skills (c), and all three scores combined (d).

This study evaluated the performance of the web-delivered BRISC (full and mini versions) in identifying emotional dysregulation, a hallmark of clinical status in patients with a range of psychiatric and neurological conditions. The study results were consistent across the full- and mini-BRISC versions. For the three BRISC scores combined, the full 45-question BRISC had a high overall accuracy of 0.93 (Fig. 3). The best classification of clinical status was at the threshold of *z* = −1.57, substantially below the population average of 0. The mini 15-question BRISC showed a similarly high accuracy of 0.92 (Fig. 4). These results support the effectiveness of the BRISC for identifying risk for a clinical disorder, manifested as loss of emotion regulation.

Negativity bias scores made the main contribution to the determination of clinical versus healthy status. For the full 45-question BRISC, the negativity bias score on its own detected clinical status best at a *z*-score of −1.14, consistent with a threshold of clinical meaningfulness. At this threshold, negativity bias scores showed high accuracy for detecting outpatients with a clinical condition. Across diagnostic categories, negativity bias scores showed the highest detection for major depressive disorder, posttraumatic stress disorder, and panic disorder. This profile of accuracy was duplicated for the mini version's negativity bias scores.

Emotional resilience and social skills separated clinical from healthy status at a higher *z*-score threshold than did negativity bias. Both emotional resilience and social skills scores showed high specificity. These scores are consistent with the view that a higher-than-average coping capacity may offset risk for a clinical condition and thus support screening and triaging decisions. Results were duplicated for the full and mini version of these scores.

These findings suggest that the BRISC functions to effectively assess the spectrum of poor through to effective emotion regulation. It provides a quick and accurate screen for identifying risk of a clinical disorder across multiple diagnostic categories that takes into account both susceptibility and coping factors. These findings support the use of the BRISC as an objective pan-diagnostic screen for multiple populations, from general through specialty. It expands on the current tools that screen for a particular diagnosis such as major depressive disorder (Mulrow et al. 1995; Rush et al. 2003). The sensitivity of the BRISC was highest in participants with diagnoses of depressive and anxiety disorders, consistent with the concept of negativity bias, but also retained a good level of classification across the other diagnostic categories. It also accomplishes the consideration of coping factors, and how they may offset risk factors, which has not been a part of previous instruments.

Strengths of the study include the large sample size, and coverage of multiple diagnostic groups. Future research is needed to extend the findings and address its limitations. The range of clinical participants included in the study was defined by the types of clinics being operated in participating sites. Future studies are needed to extend the evaluation to other diagnostic groups. Validation work with the BRISC has shown it correlates with real-world capacities such as quality of life and work productivity. Here, the cross-sectional design means there was no opportunity to follow up participants to assess the BRISC in relation to real-world functional outcomes over time. A controlled design would be of value, in which the BRISC is evaluated pretreatment and posttreatment. Future research is also needed to evaluate the replicability of the current findings, and their generalizability to additional populations. A prospective study might address this study's limitations involving the range of clinical participants and the lack of participant follow-up in relation to outcomes. Another valuable area for future studies would be to compare the sensitivity/specificity of the BRISC against multiple disorder-specific measures.

The BRISC offers a web-based tool to support the efficient management of mental and neurological health across populations. Its accuracy enables nonspecialist physicians and physician assistants to confidently screen for emotion dysregulation, as a core feature of mental health issues. The mini-BRISC offers an even briefer screen of emotional health that retains high levels of accuracy and may be especially suitable when a heavy patient load constrains the clinician's time. BRISC scores, especially negativity bias, capture maladaptive emotional reactivity to daily events and could be used to identify this feature of risk for depressive and anxiety disorders within other chronic conditions. The coping scores of emotional resilience and social skills may help to determine which patients are best able to cope with clinical issues and engage social support. Using this tool may help support early management of emotional mental health issues and limit the disproportionate flow on effects to disability and loss of productivity.

## Clinical Trial Registry

Trial Registry: http://ClinicalTrials.gov;

Registration Number: NCT00693849

URL: http://clinicaltrials.gov/ct2/show/NCT00693849

## Appendix

**Appendix 1:** BRISC items that contribute to negativity bias, emotional resilience, and social skills scores. The subset of items that define the mini version of the BRISC is indicated by bold text. Reverse scored items indicated by (–)*

Negativity bias
**I was often stressed, with my nerves on edge**
I felt I have no value as a person
I often felt annoyed at the way people treated me
**I lost hope and wanted to give up when something went wrong**
I felt out of control of my life and needed others to help me
I was reliable and could be counted on to keep my word (–)
I found it hard to wind down
I was jumpy and agitated
I found it difficult to relax
I was intolerant of anything that kept me from getting on with things
I was rather touchy
**I tended to over-react to situations**
I felt that I had nothing to look forward to
I felt that life was meaningless
**I felt down-hearted and blue**
**I felt I wasn't worth anything**
I couldn't seem to experience any positive feeling at all
I found it difficult to work up the initiative to do things
I felt I close to panic
I was worried about situations in which I might panic and make a fool of myself
Emotional resilience
**I felt very satisfied with the way I look and act**
**I responded best to positive feedback about myself**
**When receiving negative comments about myself, I looked for positive things to counter balance those comments**
I was often irritable and argued with people around me (–)
I was able to plan ahead to meet deadlines
I was proud of myself and some people might have thought I was putting myself first (–)
I was conscientious about everything I was asked to do
I was organized and could work toward a goal in a step by step way
I usually dithered around before I getting down to focusing on a task (–)
I worked hard, and always tried to achieve my goals
**There were times when people couldn't rely on me as much as they should have be able to (–)**
I usually tried to consider other people's needs and feelings
**I was always successful at completing my tasks, even if I had more tasks than others**
I was always disorganized and in a mess (–)
Social skills
**I could sense the mood of a group and discuss unspoken feelings**
I always tried to put myself into the place of those I was talking with
**I got feedback that I am a sensitive and understanding person**
**I usually took the initiative and introduced myself to strangers**
I got enormous satisfaction by getting people to like me
**I tried to build my close relationships with people**
I took part in social groups
People reacted to me as if I would do whatever it takes to get ahead (–1)
**I enjoyed socializing and chatting to other people**
I tried out exciting places and things to do
I was unable to become enthusiastic about anything (–)

* These items have been trademarked as the “BRISC”, by Brain Resource, and are available as a web-delivered assessment for clinical and research uses.

**Appendix 2:** 45-Item BRISC summary of the raw scores and their corresponding *z* and standardized 10 (STEN) scores for the composite markers; negativity bias, emotional resilience, and social skills. Raw scores were converted to standardized *z*-scores using the nonclinical norm sample of *n* = 1317*

Negativity bias (20 items)			Emotional resilience (14 items)			Social skills (11 items)		
		
Raw score	*z*-Score	STEN	Raw score	*z*-Score	STEN	Raw score	*z*-Score	STEN
20	−6.54	1	14	−6.11	1	11	−6.16	1
21	−6.44	1	15	−5.95	1	12	−5.95	1
22	−6.33	1	16	−5.78	1	13	−5.74	1
23	−6.22	1	17	−5.61	1	14	−5.53	1
24	−6.11	1	18	−5.45	1	15	−5.32	1
25	−6.01	1	19	−5.28	1	16	−5.11	1
26	−5.90	1	20	−5.12	1	17	−4.90	1
27	−5.79	1	21	−4.95	1	18	−4.69	1
28	−5.69	1	22	−4.79	1	19	−4.47	1
29	−5.58	1	23	−4.62	1	20	−4.26	1
30	−5.47	1	24	−4.46	1	21	−4.05	1
31	−5.36	1	25	−4.29	1	22	−3.84	1
32	−5.26	1	26	−4.13	1	23	−3.63	1
33	−5.15	1	27	−3.96	1	24	−3.42	1
34	−5.04	1	28	−3.79	1	25	−3.21	1
35	−4.94	1	29	−3.63	1	26	−3.00	1
36	−4.83	1	30	−3.46	1	27	−2.79	1
37	−4.72	1	31	−3.30	1	28	−2.58	1
38	−4.62	1	32	−3.13	1	29	−2.37	1
39	−4.51	1	33	−2.97	1	30	−2.16	1.2
40	−4.40	1	34	−2.80	1	31	−1.95	1.6
41	−4.29	1	35	−2.64	1	32	−1.74	2.0
42	−4.19	1	36	−2.47	1	33	−1.53	2.4
43	−4.08	1	37	−2.31	1	34	−1.32	2.9
44	−3.97	1	38	−2.14	1.2	35	−1.11	3.3
45	−3.87	1	39	−1.97	1.6	36	−0.90	3.7
46	−3.76	1	40	−1.81	1.9	37	−0.69	4.1
47	−3.65	1	41	−1.64	2.2	38	−0.48	4.5
48	−3.54	1	42	−1.48	2.5	39	−0.27	5.0
49	−3.44	1	43	−1.31	2.9	40	−0.06	5.4
50	−3.33	1	44	−1.15	3.2	41	0.15	5.8
51	−3.22	1	45	−0.98	3.5	42	0.36	6.2
52	−3.12	1	46	−0.82	3.9	43	0.57	6.6
53	−3.01	1	47	−0.65	4.2	44	0.78	7.1
54	−2.90	1	48	−0.49	4.5	45	0.99	7.5
55	−2.79	1	49	−0.32	4.9	46	1.20	7.9
56	−2.69	1	50	−0.15	5.2	47	1.41	8.3
57	−2.58	1	51	0.01	5.5	48	1.62	8.7
58	−2.47	1	52	0.18	5.9	49	1.83	9.2
59	−2.37	1	53	0.34	6.2	50	2.04	9.6
60	−2.26	1	54	0.51	6.5	51	2.25	10
61	−2.15	1.2	55	0.67	6.8	52	2.46	10
62	−2.04	1.4	56	0.84	7.2	53	2.67	10
63	−1.94	1.6	57	1.00	7.5	54	2.88	10
64	−1.83	1.8	58	1.17	7.8	55	3.09	10
65	−1.72	2.1	59	1.33	8.2			
66	−1.62	2.3	60	1.50	8.5			
67	−1.51	2.5	61	1.67	8.8			
68	−1.40	2.7	62	1.83	9.2			
69	−1.29	2.9	63	2.00	9.5			
70	−1.19	3.1	64	2.16	9.8			
71	−1.08	3.3	65	2.33	10			
72	−0.97	3.6	66	2.49	10			
73	−0.87	3.8	67	2.66	10			
74	−0.76	4.0	68	2.82	10			
75	−0.65	4.2	69	2.99	10			
76	−0.54	4.4	70	3.15	10			
77	−0.44	4.6						
78	−0.33	4.8						
79	−0.22	5.1						
80	−0.12	5.3						
81	−0.01	5.5						
82	0.10	5.7						
83	0.21	5.9						
84	0.31	6.1						
85	0.42	6.3						
86	0.53	6.6						
87	0.63	6.8						
88	0.74	7.0						
89	0.85	7.2						
90	0.95	7.4						
91	1.06	7.6						
92	1.17	7.8						
93	1.28	8.1						
94	1.38	8.3						
95	1.49	8.5						
96	1.60	8.7						
97	1.70	8.9						
98	1.81	9.1						
99	1.92	9.3						
100	2.03	9.6						

* Of these nonclinical norms, 579 were also included in this study and in each case were identified as having good brain health status.

**Appendix 3:** 15-item BRISC summary of the raw scores and their corresponding z and standardized 10 (STEN) scores for the composite markers; negativity bias, emotional resilience, and social skills. Raw scores were converted to standardized *z*-scores using the nonclinical norm sample of *n* = 1317*

Negativity bias (5 items)			Emotional resilience (5 items)			Social skills (5 items)		
		
Raw score	*z*-Score	STEN	Raw score	*z*-Score	STEN	Raw score	*z*-Score	STEN
5	−5.54	1	5	−4.91	1	5	−4.33	1
6	−5.16	1	6	−4.52	1	6	−4.00	1
7	−4.79	1	7	−4.12	1	7	−3.67	1
8	−4.42	1	8	−3.72	1	8	−3.33	1
9	−4.04	1	9	−3.33	1	9	−3.00	1
10	−3.67	1	10	−2.93	1	10	−2.67	1
11	−3.30	1	11	−2.54	1	11	−2.33	1
12	−2.92	1	12	−2.14	1.2	12	−2.00	1.5
13	−2.55	1	13	−1.74	2.0	13	−1.67	2.2
14	−2.18	1.1	14	−1.35	2.8	14	−1.33	2.8
15	−1.80	1.9	15	−0.95	3.6	15	−1.00	3.5
16	−1.43	2.6	16	−0.56	4.4	16	−0.67	4.2
17	−1.06	3.4	17	−0.16	5.2	17	−0.33	4.8
18	−0.68	4.1	18	0.24	6.0	18	0.00	5.5
19	−0.31	4.9	19	0.63	6.8	19	0.33	6.2
20	0.06	5.6	20	1.03	7.6	20	0.67	6.8
21	0.44	6.4	21	1.42	8.3	21	1.00	7.5
22	0.81	7.1	22	1.82	9.1	22	1.33	8.2
23	1.18	7.9	23	2.22	9.9	23	1.67	8.8
24	1.55	8.6	24	2.61	10	24	2.00	9.5
25	1.93	9.4	25	3.01	10	25	2.33	10

* Of these nonclinical norms, 579 were also included in this study and in each case were identified as having good brain health status.
